# Calcineurin, Synaptic Plasticity, and Memory

**DOI:** 10.1100/tsw.2001.259

**Published:** 2001-10-11

**Authors:** Carl Weitlauf, Danny Winder

**Affiliations:** Vanderbilt University School of Medicine, Department of Molecular Physiology and Biophysics, Nasville, TN 37235, USA

**Keywords:** calcineurin, synaptic plasticity, memory, long-term potentiation (LTP)

## Abstract

A long-held hypothesis in neuroscience holds that learning and memory mechanisms involve lasting changes in synaptic weights. Multiple mechanisms for producing such changes exist, of which NMDA-receptor–dependent long-term potentiation (LTP) is the most widely studied. Curiously, the relatively simple hypothesis that LTP plays a role in learning and memory has proven difficult to test. A current experimental strategy is to generate genetically altered mice with mutations in genes thought to be involved in LTP and assess the effects of these mutations both on LTP and animal behavior[1,2]. A difficulty associated with these approaches has been that they are not temporally or spatially refined. To alleviate this problem, Dr. Isabelle Mansuy and colleagues used an inducible and reversible transgene expression system in which transgene expression could be controlled on a week-to-week timescale to assess the effects of genetic reduction of the activity of a protein phosphatase known as calcineurin or PP2B in adult mouse forebrain[3,4].

Phosphorylation/dephosphorylation plays a critical role in the induction of LTP, and pharmacological and genetic approaches have begun to tease out the various roles played by specific kinases. Until recently though, little emphasis has been placed upon phosphatases. However, two key sets of pharmacological studies, two by Mulkey and Malenka[5,6] demonstrating a requirement for phosphatase activity in weakening synaptic strength, and two by Blitzer et al.[7,8] demonstrating that PKA is required for LTP induction in part to suppress phosphatase activity, have fueled interest in these enzymes. Indeed, phosphatases, once thought to play a somewhat mundane, accessory role, are starting to receive more attention as active mediators and regulators of synaptic plasticity[9]. A model first proposed by John Lisman posits that calcium influx through postsynaptic NMDA receptors is the first step in the induction of LTP[10]. In this model, strong synaptic activity would lead to large increases in intracellular calcium, resulting in activation of CaMKII, a key kinase required for LTP induction, and protein kinase A (PKA). This model postualted that CaMKII activation would induce an increase in synaptic strength, in collaboration with PKA-mediated suppression of phosphatase activity. A number of studies have provided evidence in support of aspects of this model (for review see [9]).

The phosphatase calcineurin has begun to receive attention due to its regulation by calcium. Previous studies have addressed the role of calcineurin in hippocampal synaptic plasticity through the use of pharmacological inhibitors. Curiously, the results of these studies have been somewhat inconsistent. Pharmacological inhibition of calcineurin has been reported to attenuate[11,12,13], enhance[14], or have no effect[6] on NMDA-receptor–dependent LTP. These differences likely reflect a combination of issues, including differences in plasticity induction protocols, animal age, and lack of specificity of some calcineurin inhibitors, as well as the pleiotropic roles of this phosphatase. Genetic approaches have also been taken to modulate calcineurin activity in the brain in an effort to gain further insight into the role of calcineurin in synaptic plasticity, as well as to determine the behavioral roles of this enzyme. In initial genetic studies, it was shown that mice which inducibly overexpress an active calcineurin transgene in forebrain have reduced hippocampal CA1 LTP accompanied by learning deficits[3,15,16]. Conversely, antisense oligonucleotides have been utilized to reduce calcineurin catalytic subunit expression levels in hippocampus of rat, which results in enhanced LTP as well as enhanced performance of the rats in a contextual fear-conditioning task[17,18]. Thus, these studies indicate a role for the phosphatase in constraining both LTP and memory across species.

These findings were recently further extended by results reported in Malleret et al.[4]. In this study, genetic inhibition of endogenous calcineurin via inducible and reversible overexpression of the autoinhibitory domain of the enzyme in mice resulted in enhanced performance in learning and memory tasks, as well as a corresponding increase in hippocampal LTP observed both *in vivo* and *in vitro*.

To assess the effect of regulation of forebrain calcineurin activity on learning and memory, this study focused primarily on a novel object recognition task, as well as the Morris water maze. In the novel object recognition task the mouse is introduced to three objects in a box, following which (at increasing retention intervals) the mouse is re-exposed to the box where either the location of the objects has been altered, or an object has been replaced with a novel object. An increased percentage of time spent exploring the novel or misplaced object compared to the others in the box is thought to be indicative of recognition that this object or location is "newer" than the others. In the "simple" training regimen employed, wildtype mice "recognized" the novel object as assessed by a discrimination ratio 24 h after training, but this discrimination disappeared 3 days to 1 week afterwards. Mice overexpressing the autoinhibitory domain of calcineurin exhibited a similar discrimination ratio 24 h after training, but in contrast to wildtype mice, clear discrimination was still evident 1 week after training. These findings were corroborated by studies in the Morris water maze. In this task, mice are taught to escape from an opaque pool of water to a submerged platform based on fixed distal visual cues. Once again, mice overexpressing the autoinhibitory domain of calcineurin exhibited improved performance, as the latencies with which these mice found the hidden platform were shorter than those of controls, even though swim speed was unchanged. *In vivo* electrophysiological recordings from the hippocampi of awake as well as anesthetized mutant mice revealed enhanced LTP in the dentate gyrus and area CA1 for a period exceeding 3 days in some cases. Enhancement of LTP was also observed *in vitro* with extracellular field recordings from area CA1. The *in vitro* studies revealed that the enhanced LTP was NMDA receptor dependent, and blocked by a PKA inhibitor, providing initial clues to the mechanism of action of calcineurin. Importantly, both the memory improvements and plasticity enhancements were abolished when the mutations were reversed, showing that the effects were due to transient changes in calcineurin activity and not lasting changes in the brain.

Thus, through the use of genetic approaches, accumulating evidence supports a role for calcineurin as a negative regulator of both hippocampal-based learning and LTP. It should be pointed out, however, that as with the pharmacological studies, genetic studies suggest that the role of calcineurin in synaptic plasticity is likely dependent on the state of the synapse and the pattern of synaptic activity that recruits plastic processes, as phenotypes are observed with some protocols but not others in these mice[4,15,19,20]. Thus, it is increasingly clear that a complete understanding of the role of this enzyme in plasticity, learning and memory will require the identification of the substrates dephosphorylated by this enzyme during these processes. Indeed, much work in the field currently focuses on the substrates of kinases and phosphatases involved in plasticity and learning, in particular through the use of phosphorylation site-specific antibodies. Combining these approaches with mutant mice such as those described here should aid not only in our determination of the specific roles of these signaling enzymes in synaptic plasticity, but also whether it is these roles that actually underlie the effects of manipulation of these enzymes on learning and memory.
